# The Role of Acid-Sensing Ion Channel 1A (ASIC1A) in the Behavioral and Synaptic Effects of Oxycodone and Other Opioids

**DOI:** 10.3390/ijms252111584

**Published:** 2024-10-29

**Authors:** Margaret J. Fuller, Noah R. R. Andrys, Subhash C. Gupta, Ali Ghobbeh, Collin J. Kreple, Rong Fan, Rebecca J. Taugher-Hebl, Jason J. Radley, Ryan T. Lalumiere, John A. Wemmie

**Affiliations:** 1 Department of Psychiatry, University of Iowa, Iowa City, IA 52242, USA; margaret.fuller@hsc.utah.edu (M.J.F.); noah-andrys@uiowa.edu (N.R.R.A.); subhash-gupta@uiowa.edu (S.C.G.); rebecca-taugher@uiowa.edu (R.J.T.-H.); 2 Department of Veterans Affairs Medical Center, Iowa City, IA 52242, USA; 3 Department of Molecular Physiology and Biophysics, University of Iowa, Iowa City, IA 52242, USA; 4 Medical Scientist Training Program, University of Iowa, Iowa City, IA 52242, USA; 5 Department of Psychiatry, University of Utah, Salt Lake City, UT 84112, USA; 6 Department of Neurology, University of Wisconsin School of Medicine and Public Health, Madison, WI 53726, USA; 7 Department of Psychological and Brain Sciences, University of Iowa, Iowa City, IA 52242, USA; jason-radley@uiowa.edu (J.J.R.); ryan-lalumiere@uiowa.edu (R.T.L.); 8 Iowa Neuroscience Institute, University of Iowa, Iowa City, IA 52242, USA; 9 Interdisciplinary Graduate Program in Neuroscience, University of Iowa, Iowa City, IA 52242, USA; 10 Department of Neurosurgery, University of Iowa, Iowa City, IA 52242, USA

**Keywords:** ASIC, ASIC1A, oxycodone, opioid, withdrawal, dendritic spine, nucleus accumbens, substance use disorder, opioid use disorder, AMPAR/NMDAR

## Abstract

Opioid-seeking behaviors depend on glutamatergic plasticity in the nucleus accumbens core (NAcc). Here we investigated whether the behavioral and synaptic effects of opioids are influenced by acid-sensing ion channel 1A (ASIC1A). We tested the effects of ASIC1A on responses to several opioids and found that *Asic1a^−/−^* mice had elevated behavioral responses to acute opioid administration as well as opioid seeking behavior in conditioned place preference (CPP). Region-restricted restoration of ASIC1A in NAcc was sufficient to reduce opioid CPP, suggesting NAcc is an important site of action. We next tested the effects of oxycodone withdrawal on dendritic spines in NAcc. We found effects of oxycodone and ASIC1A that contrasted with changes previously described following cocaine withdrawal. Finally, we examined α-amino-3-hydroxy-5-methyl-4-isoxazolepropionic acid (AMPA) receptor-mediated and N-methyl-D-aspartic acid (NMDA) receptor-mediated synaptic currents in NAcc. Oxycodone withdrawal, like morphine withdrawal, increased the AMPAR/NMDAR ratio in *Asic1a^+/+^* mice, whereas oxycodone withdrawal reduced the AMPAR/NMDAR ratio in *Asic1a^−/−^* mice. A single dose of oxycodone was sufficient to induce this paradoxical effect in *Asic1a^−/−^* mice, suggesting an increased sensitivity to oxycodone. We conclude that ASIC1A plays an important role in the behavioral and synaptic effects of opioids and may constitute a potential future target for developing novel therapies.

## 1. Introduction

Opioid use disorder (OUD) is a major public health challenge in the United States, with overdose deaths exceeding 100,000 in 2023 [1]. Though long-term opioid replacement therapy has improved outcomes for many patients, mortality remains high [2]. New knowledge about the brain mechanisms of addiction may lead to novel therapies to address the gaps in treatment. Dendritic spines constitute postsynaptic contacts for glutamatergic synapses, and drugs of abuse are known to alter the morphology of spines in key addiction-relevant brain regions including NAcc [3]. Drugs of abuse also alter glutamatergic transmission, including effects on the AMPAR/NMDAR ratio, which is related to synaptic strength [4]. Changes in dendritic spine morphology and AMPAR/NMDAR ratio in the NAcc may be critical for the development of substance use disorders and relapse in withdrawal [5], including to opioids [4,6,7]. Opioid effects on glutamatergic transmission in NAcc medium spiny neurons (MSNs) are necessary for normal opioid self-administration and CPP [7,8,9,10,11,12,13]. Oxycodone has played a significant role in the opioid crisis, and prescription opioid use is a major risk factor for the use of illicit opioids like heroin [14,15,16]. Despite its clinical relevance, oxycodone is less well-studied than other opioids. As such, the effects of oxycodone withdrawal on dendritic spines and AMPAR/NMDAR ratio in the NAcc have not been determined. We, therefore, focused much of the present study on investigating the synaptic effects of oxycodone.

Acid-sensing ion channels (ASICs) are cation channels that are present in the NAcc that have been implicated in rodent behaviors induced by drugs of abuse. These channels produce a current at postsynaptic sites in the NAcc in response to extracellular reductions in pH that occur during neurotransmission [17]. Channels are composed of several subunits present in the brain, ASIC1A, ASIC2A, and ASIC2B [18]. Of these, ASIC1A is the most abundant and is required for the normal pH sensitivity of the channel [19,20,21]. Disruption of ASIC1A reduced the locomotor activity to acute cocaine injection and increased CPP to both cocaine and morphine [20,22]. Our previous work also suggested that ASIC1A has a role in dendritic spine morphology and glutamatergic transmission in the NAcc, and its disruption may lead to increased sensitivity to the effects of cocaine [20]. ASIC1A interacts with a number of molecular mediators involved in the synaptic effects of drugs of abuse. For example, ASIC1A binds to Protein Interacting with C Kinase 1 (PICK1), a protein in the brain required for normal reinstatement of cocaine self-administration [23], and this interaction increases ASIC1A localization at synapses [24,25]. PICK1 also modulates dendritic spine morphology and AMPAR subunit composition [26,27]. These interactions may contribute to the role of ASIC1A in the synaptic effects of cocaine. However, the role of ASIC1A in behavioral and synaptic responses to opioids has not been fully elucidated. While drug seeking to both cocaine and morphine is elevated in *Asic1a^−/−^* mice, it is not known if these behavioral observations share a common underlying mechanism.

In dorsal root ganglion neurons, both morphine and another opioid receptor agonist, (D-Ala^2^, *N*-MePhe^4^, Gly-ol)-enkephalin (DAMGO), reduced the ASIC current by half. These effects were blocked by naloxone, suggesting they were mediated by binding to the mu opioid receptor [28]. Interestingly, the endogenous opioids dynorphin A and big dynorphin decreased the sensitivity of ASIC1A to steady-state desensitization, which may promote increased charge transfer through ASIC1A [29]. Thus, ASIC1A may have a role in opioid responses that differs from its involvement in cocaine effects.

ASIC1A plays a role in both acute responses and conditioned behaviors driven by drugs of abuse. However, the role of ASIC1A in the synaptic effects of opioids has not been previously assessed. We, therefore, investigated the consequences of ASIC1A disruption on behavior and synaptic effects driven by a number of different opioids including oxycodone. Here we identified roles for ASIC1A in responses to opioids, which in comparison to the previously described effects of cocaine, suggest distinct effects of ASIC1A across diverse drug classes.

## 2. Results

**Figure 1:**
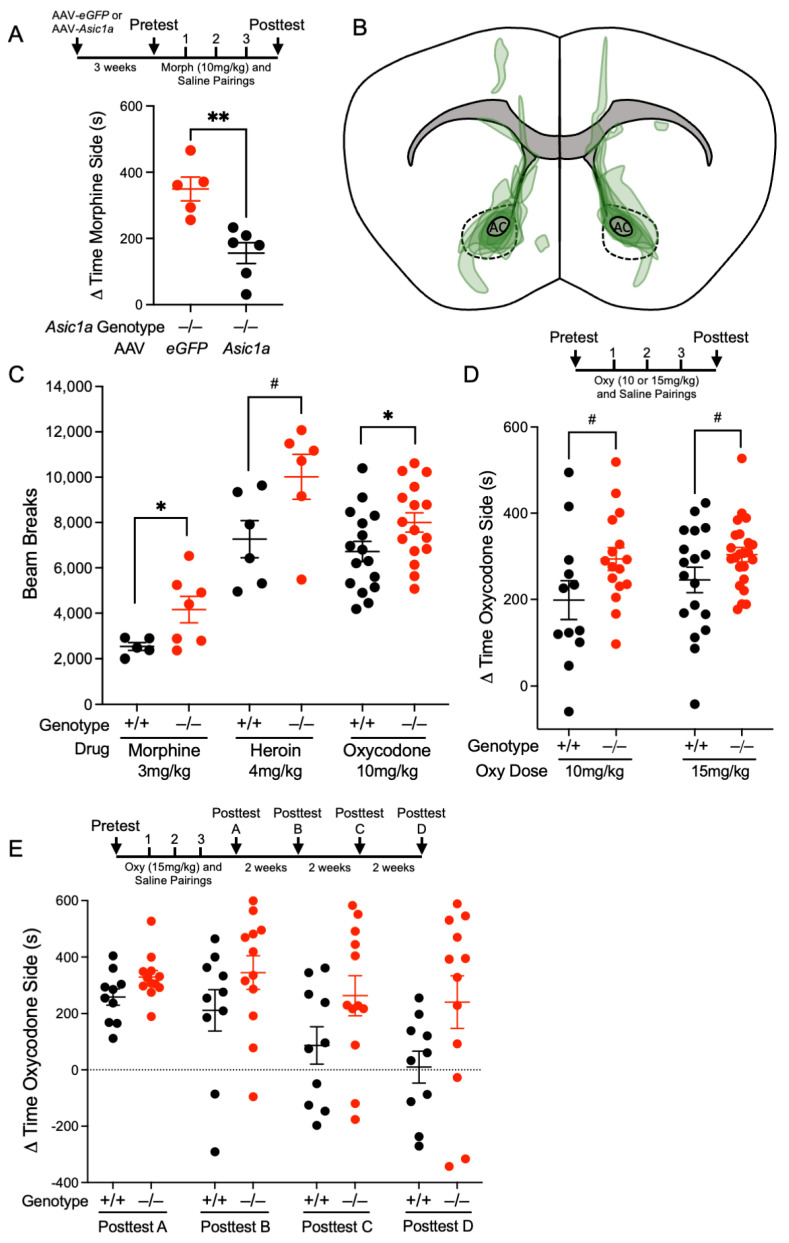
ASIC1A disruption increased behavioral responses to multiple opioids, while restoring ASIC1A in NAcc of *Asic1a^−/−^* mice attenuated morphine CPP. (**A**) AAV-*eGFP* and AAV-*Asic1a* were injected into bilateral NAcc via stereotactic injection in *Asic1a^−/−^* mice and morphine CPP was performed. (**B**) Map of viral targeting verification in slices of AAV-*Asic1a*-injected morphine CPP mice. AC = anterior commissure. Dotted lines encircle NAcc. (**C**) Locomotor activity (Beam Breaks) in *Asic1a^−/−^* and *Asic1a^+/+^* mice following acute injection of morphine, heroin, and oxycodone. (**D**) Oxycodone CPP at two different oxycodone doses. (**E**) Oxycodone CPP and withdrawal tests to assess maintenance of side preference. Dotted horizontal line shows y = 0.0 at which there was no difference between pre-test and post-test time spent on the oxycodone-paired side. Significance bars indicate *p*-values for *t*-tests (*p* ≤ 0.05 designated *, *p* ≤ 0.01 designated **, *p* ≤ 0.10 designated #).

To determine the role of ASIC1A in opioid-evoked behaviors, we assessed the effect of ASIC1A disruption on conditioned place preference and open field behavior. Our previous work revealed that *Asic1a^−/−^* mice had increased CPP to cocaine and that the NAcc was a key site of action for ASIC1A in this behavior [20]. We delivered an adeno-associated virus (AAV) containing *Asic1a* to the NAcc bilaterally in *Asic1a^−/−^* mice and tested CPP to morphine. In our previous work, this method induced enough ASIC1A expression in the NAcc to produce a channel current equivalent to that of wild-type animals [20]. Mice were each given three injections of morphine and saline across three days of training, and the difference in place preference between pre-test and post-test was determined. ASIC1A transduction in the NAcc was sufficient to significantly reduce morphine CPP [*t* (9.00) = 4.070, *p* = 0.0028, Student’s *t*-test, *n* = 5 and 6 mice] (Figure 1A,B). The degree of morphine CPP was closely analogous to that previously observed in *Asic1a*^+/+^ mice using the same protocol [20]. This result suggests that the NAcc is a key site of action for ASIC1A in opioid-reinforced behavior.

Previous work indicated that locomotor responses in *Asic1a^+/+^* and *Asic1a^−/−^* mice do not differ following acute saline injection but differ significantly following acute injection of cocaine [22]. We, therefore, hypothesized that ASIC1A disruption would alter locomotor responses to acute opioid administration. Injection of morphine [*t* (7.01) = 2.682, *p* = 0.0314, Welch’s *t*-test, *n* = 5 and 7 mice], heroin [*t* (10.00) = 2.138, *p* = 0.0582, Student’s *t*-test, *n* = 6 and 6 mice], and oxycodone [*t* (30.00) = 2.076, *p* = 0.0466, Student’s *t*-test, *n* = 16 and 16 mice] intraperitoneally (i.p.) produced greater locomotor activity in the open field test in *Asic1a^−/−^* mice compared to *Asic1a^+/+^* (Figure 1C). Oxycodone responses were independent of sex via a two-way analysis of variance (ANOVA) (Figure S1A). Previous experiments found that *Asic1a^−/−^* mice had reduced locomotor activity in an open-field test following the injection of cocaine [22], suggesting that the effects of ASIC1A disruption depend on drug class. Because ASIC1A disruption increased CPP to both cocaine and morphine [20], we hypothesized that CPP would also be elevated to oxycodone. We performed oxycodone CPP and assessed the change in place preference after training (Figure 1D). ASIC1A disruption increased CPP to 10 mg/kg of oxycodone and 15 mg/kg of oxycodone (Figure 1D). There was an effect of genotype via a two-way ANOVA in oxycodone side preference but no effect of oxycodone dose in this experiment and no interaction [F (1, 65) = 7.526, *p* = 0.0079 genotype effect; F (1, 65) = 1.027, *p* = 0.3147 dose effect; F (1, 65) = 0.4270, *p* = 0.5158 interaction, *n* = 12–23 mice]. *t*-tests revealed trends in the side preference between genotypes for both doses of oxycodone [*t* (26.00) = 1.935, *p* = 0.0640, 10 mg/kg oxycodone dose, Student’s *t*-test, *n* = 12 and 16 mice; *t* (27.33) = 1.737, *p* = 0.0936, 15 mg/kg oxycodone dose, Welch’s *t*-test, *n* = 18 and 23 mice]. There was no effect of sex via a two-way ANOVA (Figure S1B). We next tested the effect of ASIC1A disruption on CPP across six weeks of withdrawal (Figure 1E). *Asic1a^−/−^* mice had more persistent oxycodone place preference than *Asic1a^+/+^* mice. There was an effect of genotype and of time via a two-way ANOVA across 4 post-tests performed during withdrawal [F (1, 20) = 4.400, *p* = 0.0489 genotype effect; F (3, 60) = 6.746, *p* = 0.0005 time effect; F (3, 60) = 1.164, *p* = 0.3310 interaction, *n* = 10 and 12 mice]. Place preference was reduced to zero by the final timepoint in *Asic1a^+/+^* mice [*t* (9.00) = 0.1735, *p* = 0.8661, a one-sample *t*-test compared to zero, *n* = 10 mice] but remained above zero for *Asic1a^−/−^* mice throughout the tested withdrawal period [*t* (11.00) = 2.577, *p* = 0.0257, a one-sample *t*-test compared to zero, *n* = 12 mice]. Together, these results suggest that ASIC1A plays a role in multiple drug-evoked behaviors to multiple opioids and that the NAcc is a key site of action.

**Figure 2:**
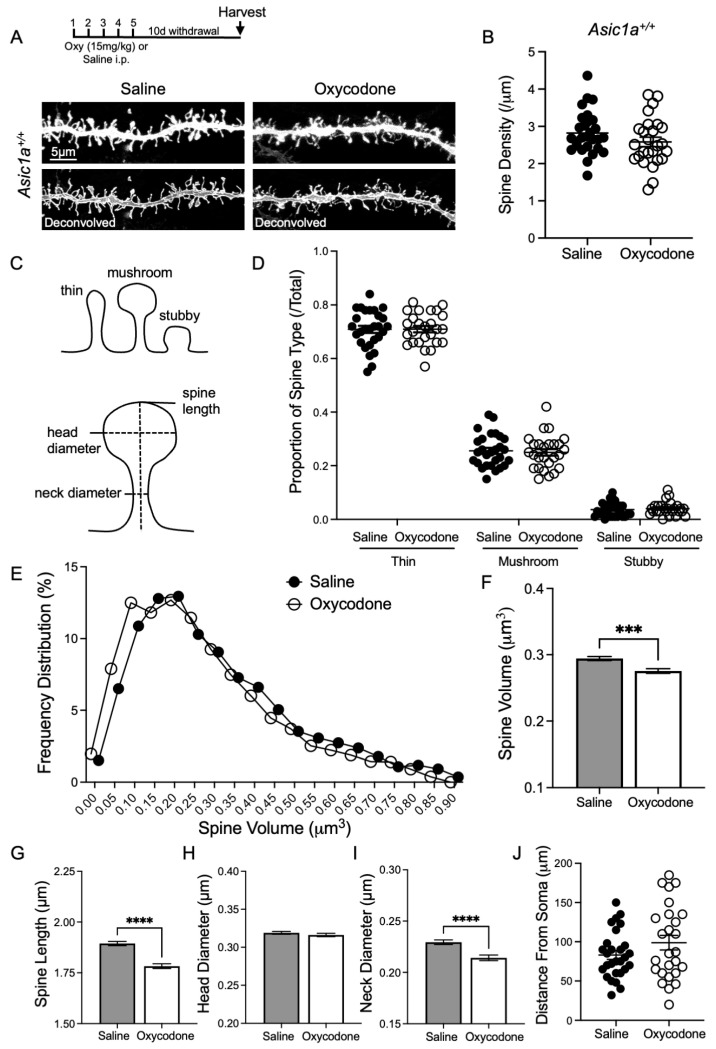
Oxycodone withdrawal significantly reduced spine volume but had no effect on spine density or proportion of spine types in *Asic1a^+/+^* mice. (**A**) Timeline of oxycodone administration and withdrawal, followed by harvesting. Representative images of NAcc MSN dendrites from *Asic1a^+/+^* mice after saline injections or oxycodone withdrawal. Top images are before deconvolution, bottom images are deconvolved. (**B**) Spine density in saline-injected and oxycodone-withdrawn *Asic1a^+/+^* mice. (**C**) Graphical illustrations of spine type classifications thin, mushroom, and stubby and of spine length, head diameter, and neck diameter. (**D**) Proportions of different spine types in saline-injected and cocaine-withdrawn *Asic1a^+/+^* mice. (**E**) Frequency distribution of spine volume in saline-injected and cocaine-withdrawn *Asic1a^+/+^* mice. (**F**) Spine volume, (**G**) spine length, (**H**) head diameter, (**I**) and neck diameter are shown as mean with standard error. (**J**) Distance from soma of each dendrite sampled. Significance bars indicate *p*-values for *t*-tests (*p* ≤ 0.001 designated ***, *p* < 0.0001 designated ****).

Previous work demonstrated a role for ASIC1A in dendritic spine morphology, which is implicated in drug-reinforced behaviors, but little is known about the effects of oxycodone on dendritic spines. Withdrawal from opioids, like other drugs of abuse, is thought to alter dendritic spine morphology in NAcc MSNs. While studies examining effects on spine density report mixed results, two studies reported increased spine density in the NAcc following morphine withdrawal in mice [30,31]. Dendritic spine morphology in the NAcc may also be altered by opioid exposure. Acute morphine administration increased mushroom-type spines in mice [32], and heroin withdrawal and extinction training in rats reduced spine head diameter in NAcc MSNs [33]. Crucially, no previous studies have examined the effects of oxycodone withdrawal on dendritic spine density or morphology in the NAcc. Opioid effects on dendritic spines have been assessed using a wide range of withdrawal schedules, up to at least two months [30], but most studies examine effects between one and two weeks of withdrawal [32,34,35,36]. We chose to examine oxycodone withdrawal at a time point within this range. Mice were administered oxycodone or saline injections for 5 days and were allowed to withdraw for 10 days (Figure 2A). Oxycodone withdrawal had no effect on spine density in *Asic1a^+/+^* mice [*t* (52.00) = 1.451, *p* = 0.1527, Student’s *t*-test, *n* = 28 and 26 dendrites] (Figure 2B). Likewise, we found no effect on the proportion of different spine morphologic types in *Asic1a^+/+^* mice via a two-way ANOVA [F (1156) = 0.0034, *p* = 0.9537 drug effect; F (2156) = 2167, *p* < 0.0001 spine type; F (2156) = 0.1048, *p* = 0.9005 interaction, *n* = 28 and 26 dendrites] (Figure 2C,D). We also assessed spine volume and other morphologic characteristics. Oxycodone withdrawal significantly reduced spine volume, which is evident in the analysis of the frequency distribution (*p* = 0.0077, Kolmogorov–Smirnov test, *n* = 3905 and 2647 spines) (Figure 2E) and mean with standard error (*p* = 0.0004, Mann–Whitney test) (Figure 2F). This effect was largely driven by reduced spine length (*p* < 0.0001, Mann–Whitney test) and narrowing of neck diameter (*p* < 0.0001, Mann–Whitney test), rather than a change in head diameter (*p* = 0.2852, Mann–Whitney test) (Figure 2G–I). Frequency distribution plots for individual spine characteristics are provided in Supplementary Figure S2. Changes in spine length and neck diameter are thought to impact synaptic function [37,38], and this consequence of oxycodone may be related to its effects on behavior in withdrawal. Dendritic segments for this analysis were sampled by an experimenter blinded to condition, and post hoc analysis indicated that samples from each group were taken from a similar distance away from the neuron soma [*t* (41.69) = 1.442, *p* = 0.1568, Welch’s *t*-test, *n* = 28 and 26 dendrites] (Figure 2J). Together these data suggest that oxycodone withdrawal reduces spine volume without affecting spine morphologic type or spine density.

**Figure 3:**
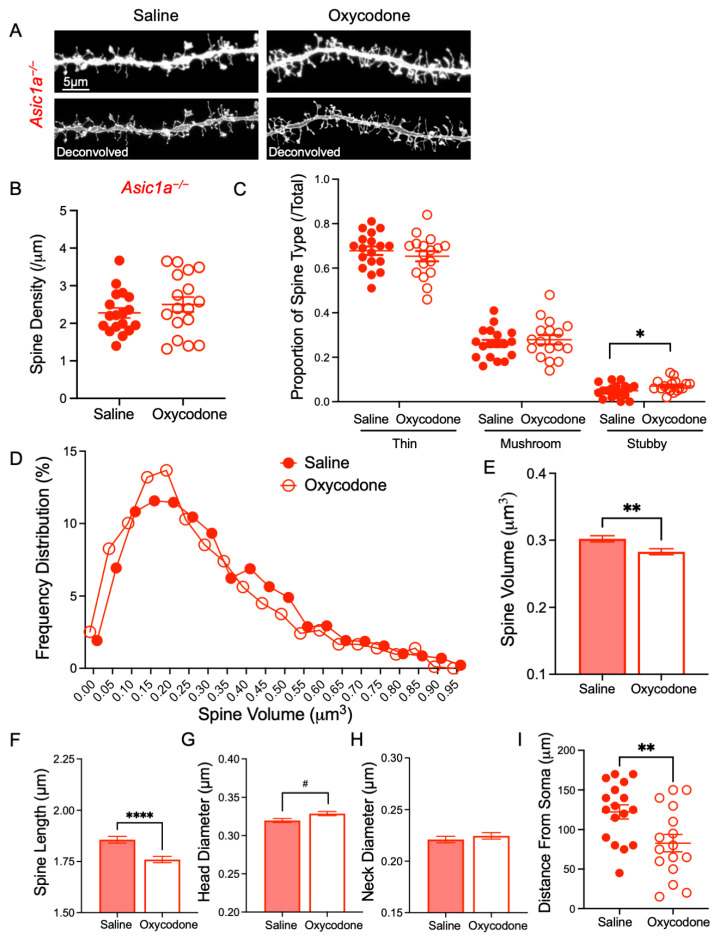
Oxycodone withdrawal had no effect on spine density and an isolated effect on the proportion of stubby spines in *Asic1a^−/−^* mice but significantly reduced spine volume. (**A**) Representative images of NAcc MSN dendrites from *Asic1a^−/−^* mice after saline injections or oxycodone withdrawal. Top images are before deconvolution, bottom images are deconvolved. (**B**) Spine density in saline-injected and oxycodone-withdrawn *Asic1a^−/−^* mice. (**C**) Proportions of different spine types in *Asic1a^−/−^* mice. (**D**) Frequency distribution of spine volume in *Asic1a^−/−^* mice. (**E**) Spine volume, (**F**) spine length, (**G**) head diameter, and (**H**) neck diameter are shown as mean with standard error. (**I**) Distance from soma of each dendrite sampled. Significance bars indicate *p*-values for *t*-tests (*p* ≤ 0.05 designated *, *p* ≤ 0.01 designated **, *p* < 0.0001 designated ****, *p* ≤ 0.10 designated #).

Our previous experiments found that ASIC1A disruption altered dendritic spine density and morphology in NAcc [20] and in mice lacking ASIC2A and ASIC2B subunits (*Asic2^−/−^*) cocaine withdrawal produced the opposite effects on spine morphology compared to wild-type mice [39]. That work suggested that disrupting ASIC function alters synaptic responses to withdrawal from drugs of abuse. We, therefore, hypothesized that synaptic responses to oxycodone withdrawal in *Asic1a^−/−^* mice would differ from *Asic1a^+/+^* mice. Following 5 days of oxycodone or saline injections, mice were allowed to withdraw for 10 days, and spine density and morphologic type were analyzed. In *Asic1a^−/−^* mice we found no effect of oxycodone withdrawal on total spine density in the NAcc [*t* (33.00) = 0.9611, *p* = 0.3435, Student’s *t*-test, *n* = 18 and 17 dendrites] (Figure 3A,B). There was a significant increase in stubby spines following oxycodone withdrawal [*t* (33.00) = 2.132, *p* = 0.0406, Student’s *t*-test, *n* = 18 and 17 dendrites], but no other effect on spine type proportions in *Asic1a^−/−^* mice via a two-way ANOVA [F (1, 99) = 0.1011, *p* = 0.7512 drug effect; F (2, 99) = 685.5, *p* < 0.0001 spine type; F (2, 99) = 1.161, *p* = 0.3174 interaction, *n* = 18 and 17 dendrites] (Figure 3C). This contrasts with our previous work using cocaine, which showed that cocaine withdrawal significantly altered proportions of spine types in *Asic2^−/−^* mice [39] and that acute cocaine administration rapidly altered spine type in *Asic1a^−/−^* mice [40]. We next assessed the effects on spine volume and other morphologic characteristics. Similar to results in *Asic1a^+/+^* mice, oxycodone withdrawal significantly reduced spine volume in *Asic1a^−/−^* mice, which is evident in the analysis of the frequency distribution (*p* = 0.0039, Kolmogorov–Smirnov test, *n* = 1877 and 1865 spines) (Figure 3D) and mean with standard error (*p* = 0.0025, Mann–Whitney test) (Figure 3E). This was driven largely by reduced spine length (*p* < 0.0001, Mann–Whitney test) (Figure 3F) and not by head diameter (*p* = 0.0686, Mann–Whitney test) (Figure 3G). There was no effect on neck diameter (*p* = 0.1240, Mann–Whitney test), which differs from our results in *Asic1a^+/+^* mice (Figure 3H). Frequency distribution plots for individual spine characteristics are provided in Figure S2. Interestingly, post hoc analysis revealed that dendritic segments sampled by an experimenter blinded to condition from oxycodone-withdrawn *Asic1a^−/−^* mice were significantly closer to the neuron soma than in saline-injected animals [*t* (31.00) = 2.792, *p* = 0.0089, Student’s *t*-test, *n* = 17 and 16 dendrites] (Figure 3I). Thus, sampling differences may have masked the effects of oxycodone in *Asic1a^−/−^* mice [41,42,43,44] and may suggest an effect of oxycodone withdrawal on dendritic arbor morphology in these mice. Additional experiments would be required to test this possibility. Overall, these results suggest that there are effects of oxycodone withdrawal on spines in *Asic1a^−/−^* NAcc that may differ from effects in *Asic1a^+/+^* mice. However, oxycodone effects are more subtle than cocaine effects, and further studies are needed to determine if synaptic transmission is impacted by these morphological changes.

**Figure 4:**
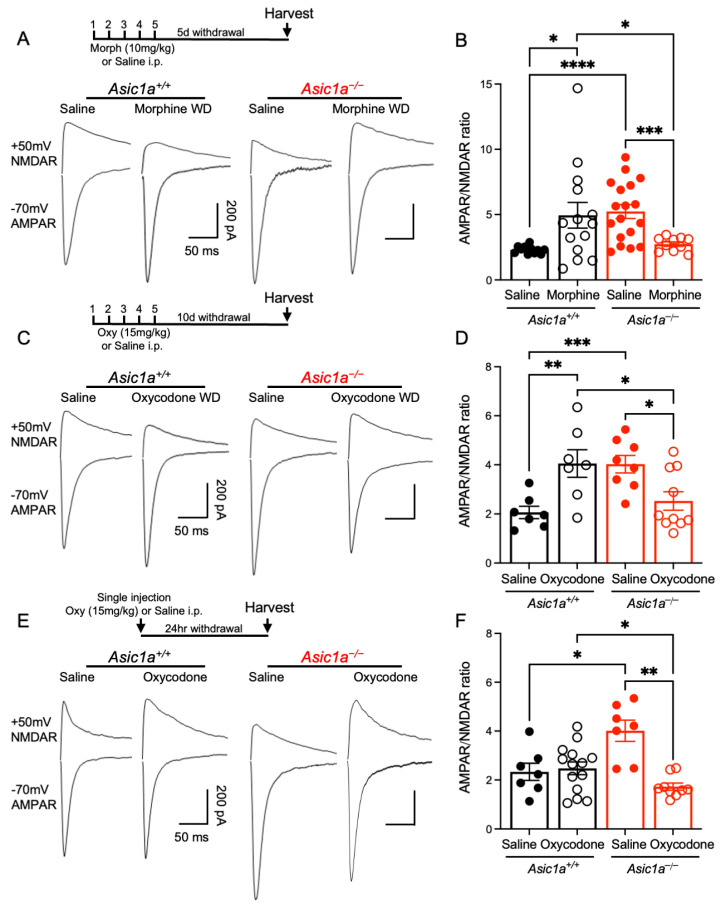
Opioid withdrawal differentially altered the AMPAR/NMDAR ratio in NAcc MSNs in *Asic1a^+/+^* versus *Asic1a^−/−^* mice. A single injection of oxycodone was sufficient to reduce the AMPAR/NMDAR ratio in *Asic1a^−/−^* mice. (**A**) Timeline of morphine administration and withdrawal, followed by harvesting. Representative traces of AMPAR-mediated excitatory postsynaptic currents (EPSCs) at −70 mV and NMDAR-mediated EPSCs at +50 mV in *Asic1a^+/+^* and *Asic1a^−/−^* mice after five daily morphine injections and five days of withdrawal. (**B**) AMPAR/NMDAR ratio following repeated saline injections or morphine withdrawal. (**C**) Timeline of oxycodone administration and withdrawal, followed by harvesting. Representative traces of AMPAR-mediated EPSCs at −70 mV and NMDAR-mediated EPSCs at +50 mV after 5 daily oxycodone injections and 10 days of withdrawal. (**D**) The AMPAR/NMDAR ratio following repeated saline injections or oxycodone withdrawal. (**E**) Timeline of a single oxycodone i.p. injection and 24 h of withdrawal, followed by harvesting. Representative traces of AMPAR-mediated EPSCs at −70 mV and NMDAR-mediated EPSCs at +50 mV 24 h after a single oxycodone injection. (**F**) The AMPAR/NMDAR ratio following one saline or one oxycodone injection. Significance bars indicate *p*-values for *t*-tests (*p* ≤ 0.05 designated *, *p* ≤ 0.01 designated **, *p* ≤ 0.001 designated ***, *p* < 0.0001 designated ****).

To explore the effects on synaptic transmission, we next assessed AMPAR and NMDAR mediated synaptic currents. AMPAR/NMDAR ratio is thought to influence drug-seeking behaviors for multiple drugs of abuse including opioids [33,45,46]. Our previous work indicated that cocaine produced paradoxical effects on AMPAR/NMDAR ratio in *Asic1a^−/−^* mice and suggested that this effect may underly altered behavioral responses to cocaine [20]. Here, we hypothesized that opioid exposure may likewise produce abnormal synaptic effects in *Asic1a^−/−^* mice. Animals were administered morphine or oxycodone i.p. for 5 days and allowed to withdraw for either 5 days or 10 days, respectively, and the AMPAR/NMDAR ratio was compared to saline-injected controls (Figure 4A). Similar to our previous work, *Asic1a^−/−^* mice exhibited an elevated AMPAR/NMDAR ratio compared to *Asic1a^+/+^* mice following saline injections [*t* (17.91) = 5.433, *p* < 0.0001, Welch’s *t*-test, *n* = 11 and 18 mice, Figure 4B; *t* (13.00) = 4.356, *p* = 0.0008, Student’s *t*-test, *n* = 7 and 8 mice, Figure 4D; *t* (12.00) = 3.011, *p* = 0.0109, Student’s *t*-test, *n* = 7 mice each, Figure 4F] (Figure 4A–F). Morphine withdrawal significantly increased the AMPAR/NMDAR ratio in *Asic1a^+/+^* mice compared to saline controls [*t* (13.21) = 2.680, *p* = 0.0187, Welch’s *t*-test, *n* = 11 and 14 mice] (Figure 4A,B). The opposite effect was seen in *Asic1a^−/−^* mice, in which morphine significantly decreased the AMPAR/NMDAR ratio compared to saline [*t* (20.24) = 4.457, *p* = 0.0002, Welch’s *t*-test, *n* = 18 and 10 mice]. This resulted in a significant difference between genotypes in the morphine-withdrawn groups by *t*-test [*t* (13.77) = 2.209, *p* = 0.0446, Welch’s *t*-test, *n* = 14 and 10 mice] and a significant interaction between drug withdrawal and genotype effects via a two-way ANOVA [F (1, 49) = 0.01337, *p* = 0.9084 drug effect; F (1, 49) = 0.3083, *p* = 0.5812 genotype effect; F (1, 49) = 15.28, *p* = 0.0002 interaction, *n* = 10–18 neurons]. Oxycodone withdrawal likewise increased the AMPAR/NMDAR ratio in *Asic1a^+/+^* mice [*t* (12.00) = 3.228, *p* = 0.0072, Student’s *t*-test, *n* = 7 mice each] and decreased this measure in *Asic1a^−/−^* mice [*t* (16.00) = 2.821, *p* = 0.0123, Student’s *t*-test, *n* = 8 and 10 mice] (Figure 4C,D). This resulted in a significant difference between genotypes in the oxycodone-withdrawn groups by *t*-test [*t* (15.00) = 2.344, *p* = 0.0332, Student’s *t*-test, *n* = 7 and 10 mice] and a significant interaction via a two-way ANOVA [F (1, 28) = 0.3663, *p* = 0.5499 drug effect; F (1, 28) = 0.2884, *p* = 0.5955 genotype effect; F (1,28) = 18.52, *p* = 0.0002 interaction, *n* = 7–10 neurons]. As with cocaine withdrawal [20], both morphine and oxycodone withdrawal produced paradoxical decreases in the AMPAR/NMDAR ratio in the *Asic1a^−/−^* NAcc. In our previous work, a single injection of cocaine was able to normalize the *Asic1a^−/−^* AMPAR/NMDAR ratio in the NAcc [20,40]. We, therefore, assessed the AMPAR/NMDAR ratio after one oxycodone injection and 24 h of withdrawal (Figure 4E). A single injection of oxycodone had no effect in *Asic1a^+/+^* mice but significantly decreased the AMPAR/NMDAR ratio in *Asic1a^−/−^* mice [*t* (7.395) = 4.981, *p* = 0.0014, Welch’s *t*-test, *n* = 7 and 9 mice] (Figure 4E,F). This resulted in a significant difference between genotypes in the oxycodone-injected groups by *t*-test [*t* (19.51) = 2.516, *p* = 0.0207, Welch’s *t*-test, *n* = 14 and 9 mice] and a significant interaction via a two-way ANOVA [F (1, 33) = 12.09, *p* = 0.0014 drug effect; F (1, 33) = 2.281, *p* = 0.1405 genotype effect; F (1, 33) = 15.52, *p* = 0.0004 interaction, *n* = 7–14 neurons]. Together, these data suggest that ASIC1A disruption alters synaptic responses in the NAcc to morphine and oxycodone. They further suggest an increased sensitivity of glutamatergic synaptic physiology to oxycodone in the *Asic1a^−/−^* NAcc. These experiments suggest an important role for ASIC1A in synaptic responses to opioids that, together with effects on dendritic spines, may underlie the elevated opioid-evoked behaviors in *Asic1a^−/−^* mice.

## 3. Discussion

Here we found that disrupting ASIC1A exacerbated multiple behavioral responses to opioids. Effects extended to multiple opioids, different doses, and across multiple timepoints of withdrawal. We also found that the NAcc is a key site of action for ASIC1A on opioid-reinforced CPP. This agreed with our previous work suggesting ASIC1A in the NAcc was sufficient to normalize cocaine CPP [20].

We further investigated potential effects of oxycodone and of ASIC1A on dendritic spines in NAcc MSNs. Previous studies reported changes in dendritic spines in response to opioids, but effects of oxycodone withdrawal on dendritic spine density and morphology in the NAcc were not known. Evidence suggests that opioids, like cocaine, alter the expression of a number of proteins involved in dendritic spine cytoskeletal structure, such as Homer1, postsynaptic density protein 95 (PSD-95), and Ras-related C3 botulinum toxin substrate 1 (Rac1) [7,47,48]. However, studies examining spine density have reported inconsistent results [30,31,32,34,36,49], and a meta-analysis found no significant effects on spine density [50]. In the present study, we investigated for the first time the effects of oxycodone withdrawal on dendritic spines in the NAcc. We found no effects on spine density per se in *Asic1a^+/+^* mice. Instead, we found effects on spine shape and size, which agrees with previous reports showing effects of heroin withdrawal on spine morphology in the NAcc [33,35]. Here we observed reduced spine volume following oxycodone withdrawal which was driven by shortening of spine length and narrowing of spine neck diameter. We did not assess the functional consequences of these changes in spine size and shape in the present study, but spine dimensions have implications for a number of important molecular and electrical processes at synapses. A shorter spine length is thought to increase biochemical and electrical coupling of the synapse with the parent dendrite [38,51]. Spine neck diameter, however, is thought to have the largest impact on function, and a narrower spine neck may reduce functional connectivity between the synapses and dendrites [37,38]. Interestingly, in a subset of NAcc MSNs, dopaminergic inputs form synapses at spine necks called triad synapses that regulate glutamatergic input at spine heads [3]. Spine neck morphology, therefore, may be an important point of functional regulation of dopamine signaling in NAcc MSNs. By altering dendritic spine size and shape, oxycodone withdrawal may produce considerable effects on synaptic function in the NAcc, and further studies are needed to examine this question directly.

ASIC1A and other ASIC subunits localize to dendritic spines and synaptosomes [52,53] and interact with a number of structural proteins essential for spine morphology [53,54,55,56,57,58,59,60]. Our previous work showed that disrupting ASIC1A altered both dendritic spine density and morphology [20], and in *Asic2^−/−^* mice, cocaine withdrawal reduced NAcc spine volume, an effect not observed in wild-type mice [39]. We hypothesized that ASIC1A disruption would lead to abnormal synaptic responses to oxycodone withdrawal. Similar to what was seen in *Asic1a^+/+^* mice, oxycodone withdrawal had no effect on spine density in the *Asic1a^−/−^* NAcc. There was a modest increase in the proportion of stubby spines following oxycodone withdrawal, but this would be unlikely to explain the elevated behavioral phenotype of *Asic1a^−/−^* mice in opioid-induced behaviors. Like *Asic1a^+/+^* mice, oxycodone withdrawal significantly reduced NAcc dendritic spine volume in *Asic1a^−/−^* mice. However, this effect was due to reduced spine length and not by neck diameter, which could suggest different functional consequences at synapses [37,38,51]. Post hoc analysis revealed that dendritic segments of oxycodone-withdrawn *Asic1a^−/−^* mice were sampled significantly closer to the neuron soma than their saline-injected counterparts. Because spine density and spine size probably differ with distance from the neuron soma [41,42,43,44], this sampling difference may have masked additional effects of oxycodone withdrawal on spine density or morphology in *Asic1a^−/−^* mice. Samples were taken preferentially from terminal dendrites farthest from the soma by an experimenter blinded to condition. Thus, reduced sampling distance from the soma could indicate a significant reduction in dendrite length or a reduction in dendritic arbor complexity, which may impact action potential propagation to the soma and filtering of postsynaptic potentials [7,61]. A previous study suggested that oxycodone CPP increased dendrite length and complexity in the NAcc and that this effect positively correlated with oxycodone CPP behavior [31]. The effect on sampling location was only seen in *Asic1a^−/−^* mice, which may suggest increased sensitivity to oxycodone withdrawal-associated morphological changes. Additional studies will be needed to directly assess dendritic arbor structure following oxycodone withdrawal. When taken together, our data suggest structural effects of oxycodone withdrawal unique to *Asic1a^−/−^* mice that may contribute to their elevated opioid-induced behaviors.

Altered AMPAR- and NMDAR-mediated glutamatergic transmission in the NAcc is thought to be critical for behaviors induced by drugs of abuse including opioids [4,6,7,62]. Whereas one study showed that withdrawal from morphine increased the AMPAR/NMDAR ratio in the nucleus accumbens shell [63,64], another study showed decreased AMPAR/NMDAR in the NAcc following extinction of heroin self-administration [33]. To our knowledge, no study has examined this measure in oxycodone withdrawal, but increased calcium-permeable AMPAR-mediated current (CP-AMPARs) in the NAcc, which is related to AMPAR/NMDAR [6], was required for incubation of oxycodone craving in withdrawal [12]. Preventing the increase in CP-AMPARs in a subset of MSNs blocked reinstatement of morphine CPP [64]. Here we found that both morphine withdrawal and oxycodone withdrawal increased the AMPAR/NMDAR ratio in the *Asic1a^−/−^* NAcc. Previous studies have suggested a correlation between increased spine head size and the AMPAR/NMDAR ratio, for example in rat hippocampal neurons [65]. However, opioid-induced plasticity may lead to differential effects on these measures [8,47]. Our data do not seem to support a correlation between head diameter and the AMPAR/NMDAR ratio, suggesting that, in the mouse NAcc and following oxycodone withdrawal, these synaptic measures are able to change independently. Interestingly, the elevated AMPAR/NMDAR ratio at baseline in *Asic1a^−/−^* mice was reduced following withdrawal, displaying a paradoxical response compared to *Asic1a^+/+^*. A single i.p. administration of oxycodone was sufficient to normalize the AMPAR/NMDAR ratio in *Asic1 a^−/−^* mice. In contrast, this same manipulation had no effect in *Asic1a^+/+^* mice. This result agrees with previous studies suggesting no effect of morphine exposure on AMPAR/NMDAR after a short withdrawal period and no effect of single injections of drugs of abuse on CP-AMPARs [6,63]. The apparent paradoxical response to oxycodone in *Asic1a^−/−^* mice on the AMPAR/NMDAR ratio parallels our previous observations with cocaine, in which a single cocaine injection reduced this measure in *Asic1a^−/−^* mice [20]. Together, these data suggest an abnormal vulnerability in *Asic1a^−/−^* mice to oxycodone-evoked changes in glutamatergic synapses in NAcc. Further, this increased sensitivity in *Asic1a^−/−^* mice appears to apply to both oxycodone and cocaine, which is consistent with elevated behavioral responses to both drugs. Some endogenous opioid peptides have been shown to modulate ASIC activity [29], but to our knowledge no studies have examined if exogenous opioidergic drugs act directly on ASICs. While this may be a possibility to assess in future studies, we posit that ASIC1a impacted opioid-induced synaptic effects here through its ability to raise intracellular Ca^2+^ via voltage-gated Ca^2+^ channels [66], thereby influencing consequences of these drugs downstream of mu opioid receptor activation.

In summary, the present study suggests a critical role for ASIC1A in multiple opioid-induced behaviors. It examines for the first time how oxycodone withdrawal alters several synaptic functions likely to mediate these behaviors and examines how ASIC1A may influence these effects. The most striking observations were the paradoxical responses in the AMPAR/NMDAR ratio to acute oxycodone exposure and oxycodone withdrawal in *Asic1a^−/−^* mice in NAcc, which may be related to their elevated behavioral responses to opioids. We speculate that enhancing ASIC1A-mediated signaling [20,66] may produce effects opposite of ASIC1A disruption, and, thus, attenuate these responses to opioids, which might be leveraged to develop new therapies for OUD.

## 4. Materials and Methods

Mice and Drugs. Male and female C57Bl6/J mice were housed in cages of no more than 5 animals on a 12 h:12 h light/dark cycle and bred in-house. Food and water were provided *ad libitum*. Experimental animals were utilized at 8–16 weeks old, and were matched within-experiment for sex and age, with no greater than a two-week age difference. *Asic1a^−/−^* mice were generated in Wemmie, Chen [67]. Morphine (Sigma-Aldrich, Burlington, MA, USA or National Institute on Drug Abuse, North Bethesda, MD, USA), oxycodone (Sigma-Aldrich, Burlington, MA, USA or National Institute on Drug Abuse, North Bethesda, MD, USA), and heroin (National Institute on Drug Abuse, North Bethesda, MD, USA) were dissolved in sterile saline and injected i.p. All procedures involving animals were approved by the University of Iowa Institutional Animal Care and Use Committee (protocol #3011094, approved 27 April 2023).

Virus Injections. Mice aged 9–13 weeks were anesthetized with ketamine/xylazine i.p. injections. Adeno-associated viruses with AAV1 capsids, cytomegalovirus promoters, and AAV2 inverted terminal repeats (AAV-*eGFP* and AAV-*Asic1a*) were produced by the University of Iowa Gene Transfer Vector Core (Iowa City, IA, USA). Mice were divided into groups and injected in bilateral NAcc similar to Kreple, Lu [20]. Briefly, 0.5 µL of either AAV-*eGFP* alone or AAV-*eGFP* and AAV-*Asic1a* mixed in a 30:70 ratio, were injected via stereotaxic injection [39,68]. Mice were left in the home cage to recover for at least three weeks before testing. Following testing, mice were sacrificed. Brains were harvested, and slices were prepared to confirm accurate viral targeting of bilateral NAcc. Any brain that did not have visible eGFP signal above background in bilateral NAcc was excluded from analysis. This resulted in the exclusion of just one animal. Verification of targeting was validated using the brain atlas Paxinos and Franklin [69].

Conditioned Place Preference. As previously described in Fuller, Gupta [39], mice were habituated to the experiment room for at least 30 min in home cages prior to each behavior session. On the first day (pre-test), animals were placed in a two-chamber CPP apparatus (Med Associates Inc., Fairfax, VT, USA) and allowed to explore both chambers freely for 20 min. The time spent on each side of the apparatus was recorded by automated sensors, and any animal that spent greater than 75% of the time on one side was excluded. On days 2–4, a panel was used to separate the two contextually distinct chambers. In the morning session, mice were weighed to determine drug dosing, and animals were injected with either a drug (10 mg/kg morphine, 10 mg/kg oxycodone, or 15 mg/kg oxycodone) or saline i.p. and placed in one side of the apparatus for 30 min. Injection and chamber were alternated for the afternoon session, and each animal received both drug and saline every day of training. On day 5 and on all subsequent post-test days, which were 2 weeks apart, animals were again allowed to explore both chambers freely for 20 min. The difference in time spent on each side of the apparatus on pre-test and post-test was calculated by simple subtraction.

Open-Field Test. Mice were habituated to the experiment room for a minimum of 30 min in home cages prior to testing. Animals were weighed to determine drug dosing and injected with 3 mg/kg morphine, 4 mg/kg heroin, or 10 mg/kg oxycodone i.p. Mice were placed in a novel open field chamber with opaque walls (San Diego Instruments, San Diego, CA, USA) and movement was recorded for 30 min by automated sensors. Boxes were cleaned between animals with glass-cleaning solution. 

Dendritic Spine Analysis. Samples were prepared for analysis and imaging was performed using a protocol similar to that described in Fuller, Gupta [39]. Briefly, mice were injected with either 15 mg/kg oxycodone or saline i.p. in the home cage for 5 days. 10 days later, they were lightly perfused by cardioperfusion with 1.5% paraformaldehyde (PFA) sodium phosphate solution (titrated to pH 7.4) under ketamine/xylazine anesthesia. Brains were harvested and placed in 1.5% PFA to continue fixation for 1–4 h. Brain slices with a thickness of 120 µm containing NAcc were prepared using a Vibratome (Vibratome 1000 Plus Sectioning System, Test Products International Inc., Beaverton, OR, USA), and small amounts of red-orange 1,1’-Dioctadecyl-3,3,3’,3’-Tetramethylindocarbocyanine Perchlorate (DiI) stain (Invitrogen, Waltham, MA, USA) were applied throughout the NAcc with a paintbrush under a dissecting microscope. Slices were then transferred to plates containing 0.01% thimerosal (Sigma-Aldrich, Burlington, MA, USA) sodium phosphate buffer solution (pH 7.4) to prevent microbial growth and placed on gentle agitation for 48 h at 4 °C. Slices were washed, and 4% PFA solution was used to fix slices before mounting with Vectashield Hardset mounting medium (Vector Laboratories, Newark, CA, USA) and 120 µm SecureSeal Imaging Spacers (Grace Bio-Labs, Bend, OR, USA). Imaging was performed on a Leica SP8 laser scanning confocal microscope (Leica Microsystems GmbH, Wetzlar, Germany) at 100× magnification using an oil-immersion objective with a 1.40 numerical aperture. Cubic voxels of 50 × 50 × 50 nm were used to acquire all images. An MSN was used for analysis only if its soma was located in the NAcc. Dendrites were preferentially selected that were terminal branches, did not overlap with other dyed segments, were filled completely with dye, and were farthest from the neuron soma. Sections of dendrite within 5 µm of a branch point or from the terminal end of the dendrite were excluded from analysis. Dendritic segments averaged 47.21 µm in length and 95.43 µm away from the neuron soma, which was measured from the most proximal point of the imaged segment. In the *Asic1a^+/+^* saline-injected group, 4122 dendritic spines were analyzed from 28 dendritic segments coming from 20 brain slices of 6 mice. In the *Asic1a^+/+^* oxycodone-withdrawn group, 2827 dendritic spines were analyzed from 26 dendritic segments coming from 13 brain slices of 6 mice. In the *Asic1a^−/−^* saline-injected group, 1976 dendritic spines were analyzed from 18 dendritic segments coming from 11 brain slices of 4 mice. In the *Asic1a^−/−^* oxycodone-withdrawn group, 1972 dendritic spines were analyzed from 17 dendritic segments coming from 10 brain slices of 4 mice. Images were deconvolved using Huygens Essential software 22.10 (Scientific Volume Imaging B.V., Hilversum, Netherlands) with automated express deconvolution settings. Dendritic segments were traced and dendritic spine morphology was analyzed using Neurolucida 360 2022.1.1 (MBF Bioscience, Williston, VT, USA) via user-guided semiautomated spine tracing. All analysis was performed with the experimenter blinded to condition. Spine morphology categories were defined similar to Fuller, Gupta [39] and Kreple, Lu [20]. Mushroom spines had head diameters > 0.35 µm and head diameter-to-neck diameter ratios >1.1:1.0. Thin spines had head diameters ≤ 0.35 µm and head diameter-to-neck diameter ratios >1.1:1.0 or head diameter-to-neck diameter ratios ≤1.1:1.0 and spine length-to-neck diameter ratios >2.5. Stubby spines had head diameter-to-neck diameter ratios ≤1.1:1.0 and spine length-to-neck diameter ratios ≤2.5. Spine volume, head diameter, neck diameter (exM neck diameter), and spine length were automatically generated by Neurolucida 360 (MBF Bioscience, Williston, VT, USA) [70]. Outlier values for these measures were removed using a ROUT test (Q = 1%), which resulted in 0.0–6.4% of values being removed from analysis in each group.

Slice Preparation for Electrophysiology. Mice were administered morphine (10 mg/kg) or oxycodone (15 mg/kg) dissolved in sterile saline, or saline only as a control, via i.p. injection in the home cage. Some animals were sacrificed after repeated administration and withdrawal, and some animals were sacrificed 24 h after a single injection. In the withdrawal condition, mice were injected daily for 5 days and allowed to withdraw for 5 days in the case of the morphine-treated groups or for 10 days in the case of the oxycodone-treated groups. Similar to previously described [39], 300 µm coronal brain sections containing NAcc were made using a Vibratome (Vibratome 1000 Plus Sectioning System, Test Products International Inc., Beaverton, OR, USA) in cold buffer. Slicing buffer solution was composed of 225 mM Sucrose, 26.0 mM NaHCO_3_, 1.20 mM KH_2_PO_4_, 1.90 mM KCl, 10.0 mM D-glucose, 1.10 mM CaCl_2_, and 2.0 mM MgSO_4_ and was continuously bubbled with 95% O_2_ and 5% CO_2_ gas. Slices were placed in 32 °C artificial cerebrospinal fluid for 30 min and then placed at room temperature for 30 min prior to recording.

Evoked Excitatory Postsynaptic Currents (EPSCs). Recordings were made as previously described [66]. Briefly, the internal recording solution contained: 125 mM cesium methanesulfonate, 20 mM CsCl, 10 mM NaCl, 2 mM Mg–adenosine 5’-triphosphate (ATP), 0.3 mM Na–guanosine 5’-triphosphate (GTP), 10 mM Hepes, 0.2 mM EGTA, and 2.5 mM QX314, pH 7.3 adjusted with CsOH. EPSCs were evoked using a bipolar tungsten electrode. The AMPAR/NMDAR ratio was calculated by dividing the evoked AMPAR-mediated EPSC amplitude at −70 mV by the NMDAR-mediated EPSC amplitude at +50 mV. NMDAR-mediated EPSCs were considered 60 ms after onset for the analysis. AMPAR- and NMDAR-mediated EPSCs were recorded in the presence of picrotoxin (100 μM). NMDAR-mediated EPSCs were recorded in the presence of cyanquixaline (CNQX, 20 μM) in the bath (all chemicals sourced from Sigma-Aldrich, Burlington, MA, USA).

Statistics. To determine differences between two groups, an unpaired two-tailed Student’s t-test was used, unless the F-test showed a significant difference in variance between groups. In this case, a Welch’s *t*-test was used. To assess differences between more than two groups, a two-way analysis of variance (ANOVA) was used. Individual *t*-tests were used in these analyses to examine a priori hypotheses of differences between two groups or differences between single groups and zero. To determine differences in distributions of two groups, as in the analysis of individual spine characteristics volume, head diameter, neck diameter, and spine length, Kolmogorov-Smirnov tests were used. Mann–Whitney tests were used to examine a priori hypotheses in these data. Sex effects were analyzed for behavior data whenever the number of animals was large enough to separate males and females. ROUT outlier tests (Q = 1%) were used in each experiment, and outliers were removed. Statistics were performed using GraphPad Prism 10, and *p*-values were considered significant if they were below 0.05.

## Data Availability

The original contributions presented in the study are included in the article/Supplementary Materials. Further inquiries can be directed to the corresponding author.

## Supplementary Materials

The following supporting information can be downloaded at: https://www.mdpi.com/article/10.3390/ijms252111584/s1.
