# Supplementary material for: The Role of Acid-Sensing Ion Channel 1A (ASIC1A) in the Behavioral and Synaptic Effects of Oxycodone and Other Opioids
Source: Int J Mol Sci. 2024 Oct 29;25(21):11584. doi: 10.3390/ijms252111584 (PMC11545886; doi:10.3390/ijms252111584)
Supplement: Supplementary file 1 [file ijms-25-11584-s001.zip › ijms-3210089-supplementary.pdf]

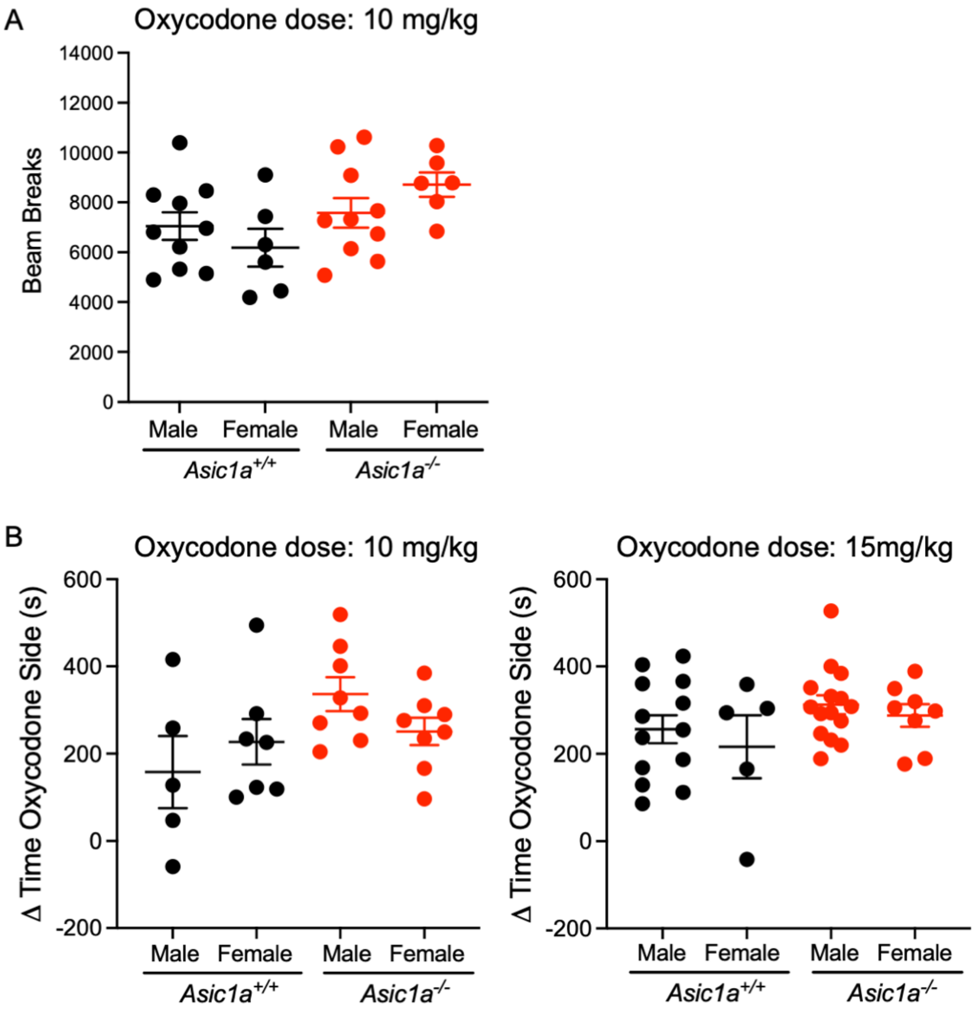

Supplementary Figure S1. There was no effect of sex on locomotor activity following acute oxycodone administration or CPP to two doses of oxycodone in *Asic1a*$^{+/+}$ and *Asic1a*$^{-/-}$ mice.

(A) Two-way ANOVA revealed no effect of sex and no interaction between sex and genotype in an open field test following acute oxycodone i.p. injection [$F(1,28) = 5.875$, $p = 0.0221$ genotype effect; $F(1,28) = 0.0457$, $p = 0.8323$ sex effect; $F(1,28) = 2.502$, $p = 0.1249$ interaction, n = 6-10 mice]. (B) Two-way ANOVA revealed no effect of sex and no interaction between sex and genotype in CPP to oxycodone using a dose of 10 mg/kg [$F(1,24) = 4.244$, $p = 0.0504$ genotype effect; $F(1,24) = 0.02845$, $p = 0.8675$ sex effect; $F(1,24) = 2.476$, $p = 0.1287$ interaction, n = 5-8 mice] or 15 mg/kg [$F(1,37) = 3.259$, $p = 0.0792$ genotype effect; $F(1,37) = 0.8337$, $p = 0.3671$ sex effect; $F(1,37) = 0.04801$, $p = 0.8278$ interaction, n = 5-15 mice].

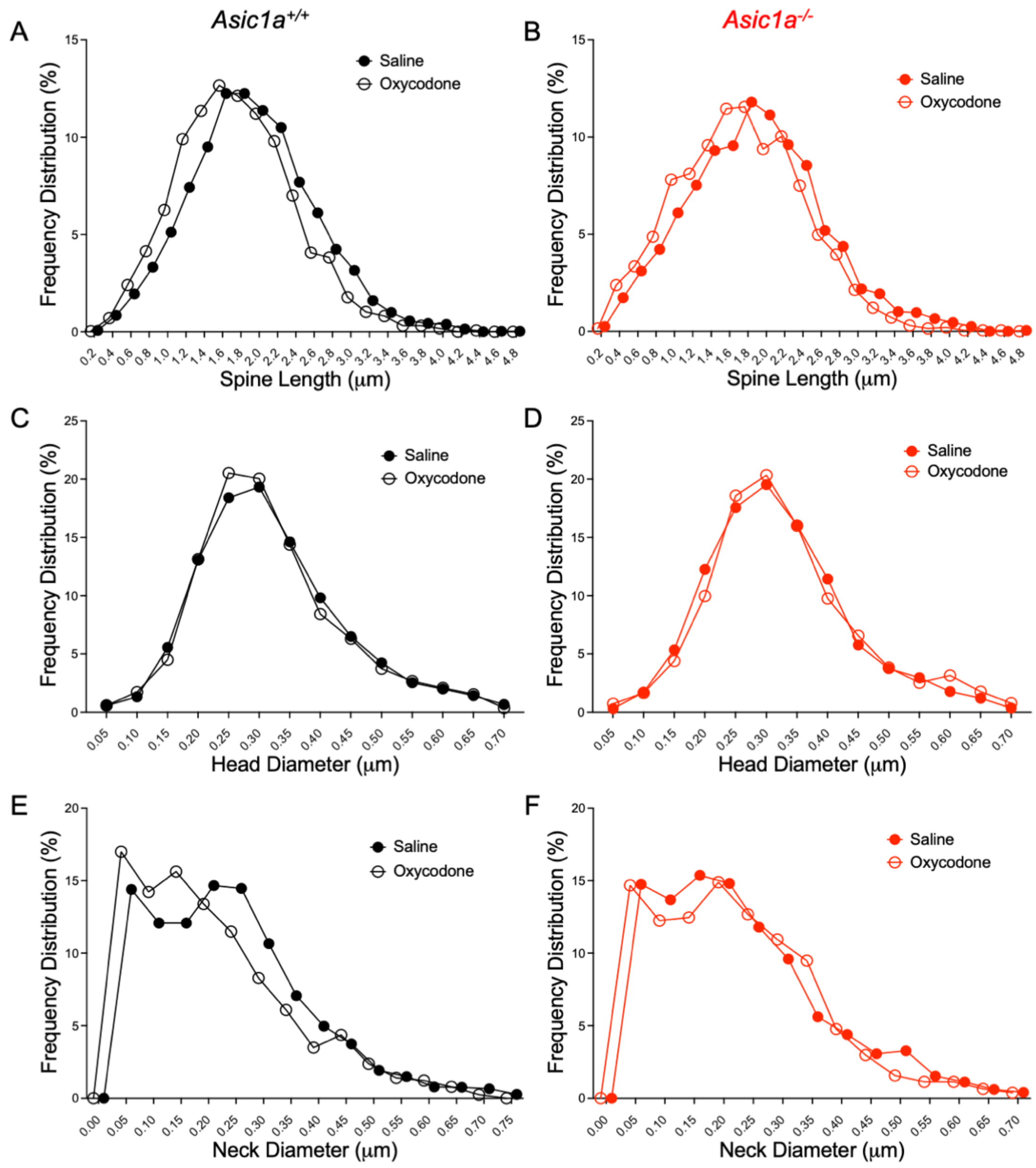

Supplementary Figure S2. Frequency distributions of spine length, head diameter, and neck diameter in *Asic1a*$^{+/+}$ and *Asic1a*$^{-/-}$ mice after oxycodone withdrawal.
(A) Oxycodone withdrawal decreased spine length in *Asic1a*$^{+/+}$ mice ($p < 0.0001$, Kolmogorov-Smirnov test, n = 4122 and 2827 spines) and (B) *Asic1a*$^{-/-}$ mice ($p = 0.0004$, Kolmogorov-Smirnov test, n = 1966 and 1972 spines). (C) No effect of oxycodone withdrawal on head diameter in *Asic1a*$^{+/+}$ mice ($p = 0.2754$, Kolmogorov-Smirnov test, n = 4035 and 2733 spines) or in (D) *Asic1a*$^{-/-}$ mice ($p = 0.2836$, Kolmogorov-Smirnov test, n = 1925 and 1937 spines). (E) Oxycodone withdrawal decreased neck diameter in *Asic1a*$^{+/+}$ mice ($p < 0.0001$, Kolmogorov-Smirnov test, n = 4091 and 2777 spines) and (F) increased neck diameter in *Asic1a*$^{-/-}$ mice ($p = 0.0492$, Kolmogorov-Smirnov test, n = 1959 and 1846 spines).
